# Evaluation of Hepatoprotective activity of *Eriocaulon quinquangulare* in vitro using porcine liver slices against ethanol induced liver toxicity and free radical scavenging capacity

**DOI:** 10.1186/s12906-016-1044-x

**Published:** 2016-02-24

**Authors:** Chamira Dilanka Fernando, Preethi Soysa

**Affiliations:** Department of Biochemistry & Molecular Biology, Faculty of Medicine, University of Colombo, Kynsey Road, Colombo, 08 Sri Lanka; College of Chemical Sciences, Institute of Chemistry Ceylon, 341/22, Kotte Road, Welikada Rajagiriya, Sri Lanka

**Keywords:** Antioxidant properties, *Eriocaulon quinquangulare*, Hepatoprotective activity, Membrane stabilization activity

## Abstract

**Background:**

Production of reactive oxygen species is a common cause in alcohol induced liver diseases. Decoction prepared from the whole plant of *Eriocaulon quinquingulare* is prescribed to treat liver disorders. The aim of this study was to investigate the hepatoprotective activity and antioxidant capacity of the water extract of *E. quinquangulare* in vitro.

**Method:**

The aqueous extract of the whole plant of *E. quinquangulare* (AEQ) was investigated for its phytochemical constituents, antioxidant and membrane stabilization properties in-vitro. The antioxidant activities of AEQ were investigated using 1,1-Diphenyl-2-picrylhydrazyl (DPPH), hydroxyl radical, nitric oxide scavenging and ferric reducing antioxidant power (FRAP) assays. Membrane stabilizing effect of the extract was determined by hypotonic solution induced human erythrocyte hemolytic assay (HEHA). Further, hepatoprotective activity against ethanol induced hepatotoxicity was carried out using porcine liver slices.

**Results:**

The total phenolics and flavonoids were 10.3 ± 1.6 w/w % gallic acid equivalents and 45.6 ± 3.8 w/w % (−)-epigallocatechin gallate equivalents respectively. The values of EC_50_ for DPPH, hydroxyl radical and nitric oxide scavenging assays were 37.2 ± 1.7 μg/ml, 170.5 ± 6.6 μg/ml and 31.8 ± 2.2 μg/ml respectively. The reducing capability of AEQ was 6.9 ± 0.2 w/w % L-ascorbic acid equivalents in the FRAP assay. For hypotonic solution induced HEHA, the IC_50_ was 1.79 ± 0.04 mg/ml. A significant decrease (*p* < 0.05) was observed in ALT, AST and LDH release from the liver slices treated with AEQ compared to the ethanol treated liver slices. A significant reduction in lipid peroxidation (*p* < 0.05) was also observed in liver slices treated with the plant extract compared to that of the ethanol treated liver slices.

**Conclusions:**

The results suggest AEQ possess hepatoprotective activity against ethanol induced liver toxicity of porcine liver slices which can be attributed to antioxidant properties and membrane stabilizing effects caused by the plant material.

**Electronic supplementary material:**

The online version of this article (doi:10.1186/s12906-016-1044-x) contains supplementary material, which is available to authorized users.

## Background

Reactive oxygen (ROS) and nitrogen species (RNS) are produced as byproducts during cellular metabolism or exposure to environment chemicals and radiation. Over production of ROS and RNS cause oxidative damage to biomolecules, provoke immune response, activate oncogenes and hasten the ageing process [1]. Chronic alcoholism leads to the development of alcoholic liver disease and is a major health problem in the society. Metabolism of ethanol occurs in the liver, catalyzed by alcohol dehydrogenase, aldehyde dehydrogenase (ALDH), cytochrome P450 2E1 (CYP2E1), and catalase enzymes [2]. These mechanisms result in the decrease of NAD^+^/NADH redox ratio and depletion of reduced glutathione (GSH) leading to oxidative stress [3, 4]. Several studies have shown that antioxidants including plant extracts protect against ethanol induced hepatotoxicity by inhibiting lipid peroxidation and enhancing antioxidant enzyme activity [5]. Use of plant derived drugs in medical practice has shown that they are relatively non-toxic, safe and free from serious side effects [6].

*Eriocaulon quinquangulare* (Family: Eriocaulaceae) locally known as “Heen kokmota” is a slender annual tuft. This monocotyledonous plant is distributed in lowlands in Sri Lanka [7]. The total plant of *Eriocaulon quinquangulare* prepared as decoction is used to treat patients suffering from liver disorders, jaundice and splenomegaly in Sri Lanka [8].

The present study was carried out to determine the phytochemical composition, antioxidant, membrane stabilization and hepatoprotective activities of aqueous extract of *E. quinquangulare* (AEQ) to evaluate the scientific base of its application as a hepatoprotective drug.

## Methods

### Chemicals and equipment

The chemicals gallic acid, Folin ciocalteu reagent, trichloroacetic acid, sodium salicylate and ethylenediamine tetra acetic acid (EDTA) were purchased from Sigma Chemicals Co. (P.O. Box 14508, St. Louis, MO 63178 USA). 1,1-Diphenyl-2-picrylhydrazyl (DPPH) free radical, (−)-epigallocatechin gallate, aluminium chloride and sulfanilamide were purchased by Fluka (Flukachemie GmbH, CH-9471 Buchs). L-ascorbic acid, hydrogen peroxide, N-(1-naphthyl)-ethylenediamine dihydrochloride and ethanol were purchased from BDH Chemicals (BDH Chemicals Ltd, Poole, England). Ferric chloride, potassium ferricyanide and sodium nitrite were purchased from Riedel De Haen Ag, Wunstorfer Strasse 40, SEELZE1, D3016, Germany.

Lactate dehydrogenase (LDH) enzyme assay kit was purchased from DiaSys (Alte Strasse 9, 65558, Holzheim, Germany). Alanine transaminase (ALT) and Aspartate transaminase (AST) enzyme assay kits were purchased from POINTE, SCIENTIFIC, INC (5449 Research Drive, Canton MI 48188, USA).

### Plant material

The whole plant of *Ericaulon quinquangulare* (Heen Kokmota) was collected from Kalutara District. This plant material was identified and confirmed by Department of Botany, Bandaranaike Memorial Ayurvedic Research Institute, Nawinna, Sri Lanka. A voucher specimen of the plant was deposited at the herbarium of the above Institute (acc number: 980c).

### Preparation of the decoction

Decoction from total plant of *Eriocaulon quinquangulare* was prepared according to a procedure followed by Ayurvedic practitioners of Sri Lanka [9]. Plant material was washed with deionized water and dried to achieve a constant weight at room temperature. Dried material was cut into small pieces and ground to a fine powder. A weight of 30 g was boiled with 800 ml of deionized water until its volume gets reduced to 100 ml (1/8^th^ of the original volume). The decoction was sonicated and filtered. The filtrate was centrifuged (2000 rpm, 10 min). The supernatant was freeze dried and stored at −20 °C in sterile tubes until further use. The yield of the lyophilized powder was calculated as a percentage of the dry weight. The lyophilized powder was dissolved in deionized water or buffer for the experiments and concentration of the treated sample was calculated as μg/ml or mg/ml of the lyophilized sample.

### Phytochemical constituents

The phytochemicals present in AEQ was determined according to a previously published method [10]. The extract was screened for carbohydrates, tannins, phenols, phlobatannins, amino acids and proteins, saponins, flavonoids, sterols, terpenoids, cardiac glycosides, alkaloids, quinones and oxalates.

### Determination of total phenolic content

Total phenolic content of AEQ was determined by Folin Ciocalteu method [11]. Calibration curve was constructed using gallic acid standards and the total phenolic content was reported as w/w% gallic acid equivalents (GAE).

### Determination of flavonoid content

The flavonoid content was quantified by the aluminium chloride colorimetric assay [11]. Calibration curve was plotted using (−)-epigallocatechin gallate (EGCG) standards and flavonoid content was reported as w/w% EGCG equivalents.

### 1,1-Diphenyl-2-picrylhydrazyl (DPPH) free radical scavenging activity

Free radical scavenging ability of AEQ prepared was assessed by DPPH radical scavenging method with slight modifications [11]. DPPH reagent prepared in absolute ethanol (100 μM, 750 μl) was added to test sample (250 μl) and the mixture was allowed to stand for 30 min in the dark. Absorbance was measured at 517 nm. Percentage inhibition was calculated according to Eq. 1

1 $$ \%\ \mathrm{Inhibition} = \frac{\mathrm{Absorbance}\ \mathrm{of}\ \mathrm{control}\ \hbox{--}\ \mathrm{Absorbance}\ \mathrm{of}\ \mathrm{sample}\ \mathrm{X}\ 100}{\mathrm{Absorbance}\ \mathrm{of}\ \mathrm{control}} $$

L-Ascorbic acid was used as the positive control. The effective concentration needed to scavenge DPPH free radical by 50 % (EC_50_) was calculated by regression analysis of the dose response curve plotted between percentage inhibition versus concentration of the test samples and the positive control.

### Hydroxyl radical scavenging activity

Hydroxyl radical scavenging activity was measured based on the competition between deoxyribose and the test compound (the plant extract) to react with hydroxyl radicals generated from Fe^2+^/Ascorbate/EDTA/H_2_O_2_ system according to the procedure as previously described with slight modifications [12]. Gallic acid was used as the positive control. The percentage scavenging of hydroxyl radical for AEQ and the positive control was calculated according to equation 1. EC_50_ was calculated as described previously.

### Nitric oxide radical (NO) scavenging activity

NO was generated from sodium nitroprusside (SNP) and NO scavenging activity of AEQ was measured based on Griess-Ilosvay reaction with slight modification [13]. The interference from the plant extract with the pink chromophore formed was minimized by background subtraction of absorbance for respective concentrations. L-Ascorbic acid was used as the positive control. The percentage scavenging of NO for AEQ and the positive control was calculated according to equation 1. EC_50_ was calculated as described previously.

### Ferric reducing antioxidant power (FRAP) assay

The ferric ion reducing power of AEQ was determined according to a method described previously [14]. L-ascorbic acid was used as the positive control. Dose response curve was plotted between the absorbance versus concentrations of plant extract or positive control. The ferric reducing antioxidant power of the decoction was expressed as w/w% L-ascorbic acid equivalents.

### Porcine liver tissue collection

Porcine liver tissue of either sex was obtained from the registered slaughter house in Dematagoda, Sri Lanka with permission obtained from the chief municipal veterinary surgeon (Refer the Section III.24 of Additional file 1 regarding ethical approval).

A sample of liver tissue without distinction of lobes was excised using sterile scalpel blades and transferred immediately into ice cold sterile Krebs Ringers-4-(2-hydroxyethyl)-1-piperazineethanesulfonic acid (HEPES) buffer (KRHB) and transported to the laboratory on the day of the experiment within 5 min in an ice bath.

### Preparation of medium

The buffer (KRHB) composition includes HEPES (2.5 mM), NaCl (118 mM), KCl (2.85 mM), CaCl_2_ (2.5 mM), KH_2_PO_4_ (1.5 mM), MgSO_4_ (1.18 mM) and glucose (4.0 mM). The pH was adjusted to 7.4 by 1 N NaOH. The medium was autoclaved for sterilization.

### Hepatoprotective activity of *E. quinquangulare* decoction in vitro

Hepatoprotective activity of AEQ was evaluated according to a method published previously using porcine liver tissue obtained from slaughter house [15]. Fresh, cleaned tissue was cut into thin slices (20–25 mg) and 100 mg was transferred into clean sample collection glass vials filled with KRHB. Vials were capped and pre-incubated for 30 min in a shaker water bath at 37 °C. Liver slices were drained carefully and transferred into separate vials with different concentrations of AEQ dissolved in KRHB (400, 1000 and 2000 μg/ml), co-exposed with ethanol (5 M) and incubated at 37 °C for 2 h. The final volume was adjusted to 3.0 ml with KRHB. Tissues were exposed to the plant extract in the absence of ethanol to assess any hepatotoxic effect induced by the plant extract itself. A control was carried out in KRHB (3.0 ml). Ethanol (5 M) was used to induce hepatotoxicity as reported in a previous study [15]. After the incubation period, the spent media were collected and the liver slices were homogenized at 4 °C. The homogenates were sonicated for 4 seconds and centrifuged at 4 °C. The supernatants and the media obtained from post incubation of tissues were assayed for alanine transaminase (ALT), aspartate transaminase (AST) and lactate dehydrogenase (LDH). The percentage cytotoxicity was calculated using Eq. 2

2 $$ \mathrm{The}\ \mathrm{percentage}\ \mathrm{cytotoxicity} = \frac{\mathrm{Enzyme}\kern0.5em \mathrm{activity}\kern0.5em \mathrm{in}\kern0.5em \mathrm{the}\kern0.5em \mathrm{medium}}{\mathrm{Total}\kern0.5em \mathrm{Enzyme}\kern0.5em \mathrm{activity}}\mathrm{X}\kern0.5em 100\kern0.5em \% $$

Where Total Enzyme activity = Enzyme activity in the medium + Enzyme activity in the tissue homogenate, Medium = Medium used for the incubation of liver tissue

The tissue homogenates were also assayed for total protein content and lipid peroxides formed [16, 17]. Standard curves were plotted using bovine serum albumin (BSA) and 1,1,3,3-tetraethoxypropane (TEP) standards respectively and amount of lipid peroxides formed was expressed as micrograms of malondialdehyde (MDA) equivalents formed per gram of protein.

### Membrane stabilization activity

The membrane stabilization activity of AEQ was determined using hypotonic solution induced human erythrocyte hemolytic assay (HEHA) according to the procedure described previously with slight modifications [18]. Fresh blood (5 ml) was collected and transferred to centrifuge tubes. The tubes were centrifuged at 2500 rpm for 5 min and the supernatant was removed. The cell suspension was washed 3–4 times with isotonic buffer (154 mM sodium chloride in 10 mM sodium phosphate buffer, pH 7.4) until the supernatant appeared clear. The volume of blood was measured and reconstituted as 40 % v/v suspension with isotonic buffer. Human erythrocyte suspension (40 % v/v, 50 μl) was mixed with hypotonic buffer (50 mM sodium chloride in 10 mM sodium phosphate buffer, pH 7.4, 1.0 ml) and plant extract prepared in isotonic buffer (100 μl). The samples were incubated for 20 min at room temperature followed by centrifugation (5000 rpm, 5 min). The absorbance of the supernatant was measured at 540 nm. Appropriate samples were constructed to measure background interferences caused by the plant extract and this reading was subtracted from the original readings. Sodium salicylate was used as the positive control. Percentage inhibition of hemolysis caused by AEQ and sodium salicylate was measured according to Eq. 3

3 $$ \%\ \mathrm{Inhibition}\ \mathrm{of}\ \mathrm{hemolysis} = \frac{\mathrm{Absorbance}\ \mathrm{of}\ \mathrm{control}\ \hbox{--}\ \mathrm{Absorbance}\ \mathrm{of}\ \mathrm{sample}\kern0.5em \mathrm{X}\ 100}{\mathrm{Absorbance}\ \mathrm{of}\ \mathrm{control}} $$

The effective concentration needed to inhibit the lysis of human erythrocytes by 50 % compared to the control (IC_50_) was calculated by regression analysis of the dose response curves.

### Statistical analysis

Triplicate measurements were obtained for each experiment unless otherwise specified. Students *T* test was performed for statistical analysis and results are presented as mean ± standard deviation (Mean ± SD). Value of *p* < 0.05 was considered as significant. Regression analysis and statistical analysis were carried out using Microsoft Excel. Calibration curves of the standards were considered as linear if R^2^ > 0.99. EC_50_ values were calculated from either linear or logarithmic dose response curves where R^2^ > 0.90.

## Results and discussion

### Extraction yield, phytochemical constituents, phenolic and flavonoid contents

Extraction yield obtained for the whole plant extract of *Eriocaulon quinquangulare* was 4.33 % as a percentage of dry weight. Results obtained for qualitative screening of phytochemicals in AEQ reveal that carbohydrates, tannins, phenols, phlobatannins, saponins, flavonoids and quinones were present in the extract where as sterols, terpenoids, amino acids and proteins, cardiac glycosides, alkaloids and oxalates were absent. Dietary polyphenolic compounds help to restore the balance between the natural antioxidants and free radicals by enhancing the activity of natural antioxidant enzymes such as superoxide dismutase (SOD), glutathione peroxidase (GPx), glutathione reductase (GR), glutathione-S-transferase (GST) and by direct scavenging of free radicals [19]. The total phenolic content of AEQ was 10.3 ± 1.6 w/w% GAE (Table 1). Studies conducted with the water extract of *Eriocaulon sexangulare* L. which belong to the same family, has yielded a total phenolic content of 88.62 ± 0.91 μg Catechin equivalents/mg which is lower compared to AEQ [20]. Flavonoids as plant derived antioxidants exhibit antimutagenic and free-radical scavenging activities. Several flavonoids namely catechin, apigenin, quercetin, naringenin, rutin, and venoruton are reported for their hapatoprotective activities [21]. Aluminium chloride colorimetric assay for flavonoids yielded total flavonoid content of 45.6 ± 3.8 w/w% (−)-epigallocatechin gallate (EGCG) equivalents for AEQ (Table 1) indicating that the plant extract is abundant in flavonoids which can exert health benefits. Studies conducted with water extract of *Eriocaulon sexangulare* has yielded a total flavonoid content of 9.57 ± 0.25 μg Rutin equivalents/mg compared to AEQ [20].

**Table 1:** Phytochemical composition of AEQ

Experiment	AEQ (*n* = 3)
Phenolic content (w/w% GAE)	10.3 ± 1.6
Flavonoid content (w/w % EGCG equivalents)	45.6 ± 3.8

### Antioxidant capacity

Oxidative stress is caused due to a variety of endogenous ROS/RNS produced during metabolic processes including ethanol metabolism and is believed to contribute significantly in the development of a number of diseases [3, 4, 22]. Therefore total antioxidant capacity of the decoction prepared was evaluated against different radical systems which include DPPH, hydroxyl and nitric oxide as well as the ability to reduce ferric ions to achieve better understanding of the specific activities possessed by AEQ.

**Fig 1:**
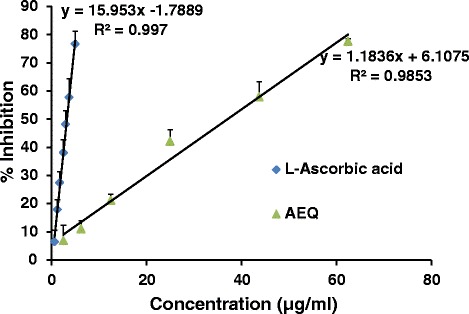
The dose response curves for percentage scavenging of DPPH by AEQ compared to L-Ascorbic acid. The results are presented as mean + SD for L-Ascorbic acid (*n* = 9) and AEQ (*n* = 3)

The hydrogen donating ability of the plant extract was determined by DPPH free radical. The free radical which is centered on nitrogen atom which is being delocalized within the aromatic system gives a characteristic purple colour measured around 517 nm. In the presence of hydrogen donors (free radical scavengers), DPPH reacts with these hydrogen atoms and forms a stable product 1,1-Diphenyl-2-picrylhydrazine resulting in a colour change from purple to yellow [23]. In the current study AEQ exhibited EC_50_ value at a concentration of 37.2 ± 1.7 μg/ml for DPPH assay which is higher than L-Ascorbic acid (3.3 ± 0.3 μg/ml) (Fig. 1). However AEQ demonstrated higher hydrogen donating ability than *Eriocaulon sexangulare* L. extract for DPPH radical scavenging assay (EC_50_ > 2000 μg/ml) [20].

**Fig 2:**
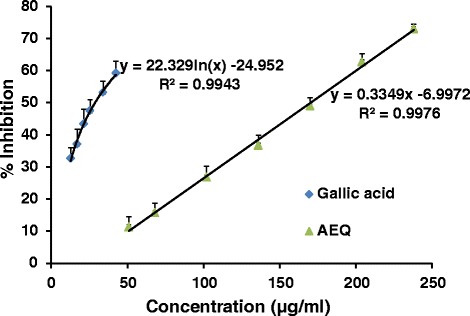
The dose response curves for percentage scavenging of hydroxyl radicals by AEQ in comparison with Gallic acid. The results are presented as mean + SD for AEQ (*n* = 3) and Gallic acid (*n* = 9)

Hydroxyl radical is an extremely reactive free radical formed in biological systems which can initiate lipid peroxidation of the cell membrane. Hydrogen peroxide generates hydroxyl radicals due to the catalysis by Iron (II). The hydroxyl radicals attack deoxyribose that eventually results in the formation of thiobarbituric acid reacting substances (TBARS) which couples with thiobarbituric acid (TBA) on heating to generate a pink-coloured product [12]. In the present study, AEQ demonstrated an EC_50_ value of 170.5 ± 6.6 μg/ml compared to gallic acid which showed an EC_50_ value of 27.7 ± 4.1 μg/ml (Table 2). The results obtained indicate the potential of the plant extract to prevent the degradation of deoxyribose in the presence of hydroxyl radicals in a dose dependent manner (Fig. 2).

**Table 2:** Antioxidant capacity of AEQ

Experiment	EC_50_ for AEQ (*n* = 3)	EC_50_ for positive control	
DPPH radical scavenging activity	37.2 ± 1.7 μg/ml	L- Ascorbic acid	3.3 ± 0.3 μg/ml (*n* = 9)
Hydroxyl radical scavenging activity	170.5 ± 6.6 μg/ml	Gallic acid	27.7 ± 4.1 μg/ml (*n* = 9)
NO scavenging activity	31.8 ± 2.2 μg/ml	L-Ascorbic acid	276.3 ± 25.8 μg/ml (*n* = 9)
FRAP assay (w/w % L-Ascorbic acid equivalents)	6.9 ± 0.2 (*n* = 3)

Nitric oxide is a small molecule that contains one unpaired electron. It is generated in biological systems by NADPH-dependent nitric oxide synthases. It acts as an important oxidative biological signaling molecule in a variety of physiological processes such as regulation of blood pressure and immune response, defense mechanism against pathogens, smooth muscle relaxation and neural signal transmission [24]. Overproduction of NO may occur when the generation of nitrogen species supersedes the ability of the biological system to neutralize and eliminate them. Increased levels of NO may lead to nitrosylation reactions that can alter the structure of protein and inhibit their function. When super oxide reacts with NO, it produces strong oxidant molecule peroxynitrite ion (ONOO^−^), which causes DNA fragmentation and lipid peroxidation [25].

**Fig 3:**
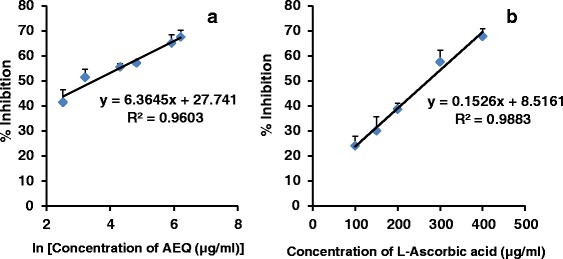
The dose response curve for percentage inhibition of NO radicals by AEQ (**a**) and L-Ascorbic acid (**b**). A linear relationship was observed between % I versus natural logarithm of concentration of AEQ. The results are presented as mean + SD of three independent experiments

Nitric oxide generated spontaneously from sodium nitroprusside (SNP) in aqueous solution at physiological pH interacts with oxygen to produce nitrite ions which in turn reacts with Griess reagent forming an azo-dye. Nitric oxide scavengers compete with oxygen which leads to reduced production of nitrite ions. The EC_50_ values obtained were 31.8 ± 2.2 and 276.3 ± 25.8 μg/ml for AEQ and L-Ascorbic acid respectively reflecting its high capacity to scavenge NO (Fig. 3).

**Fig 4:**
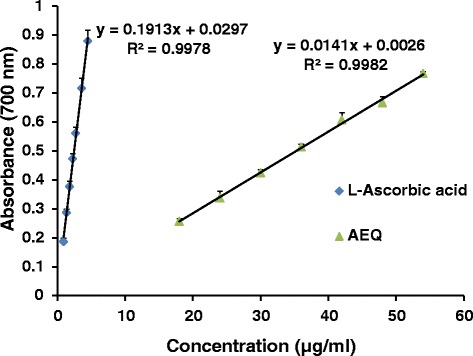
The reduction capability of different concentrations of AEQ compared to L-Ascorbic acid. The results are presented as mean + SD for AEQ (*n* = 3) and L-Ascorbic acid (*n* = 9)

FRAP can serve as an important indicator of potential antioxidant activity which involves the transformation of Fe^3+^-Fe^2+^ in the presence of the antioxidant resulting in the formation of a blue-green coloured complex [26]. The reduction capability of both the plant extract and L-ascorbic acid increased with the concentration (Fig. 4). The reducing power of AEQ was found to be 6.9 ± 0.2 w/w% L-ascorbic acid equivalents. The results obtained in the experiments for antioxidant capacity of AEQ are presented in Table 2.

### Hepatoprotective activity of AEQ in-vitro

Liver tissue obtained from slaughter house is a useful model to study hepatotoxicity of different substances in the organ level [27]. Further, liver slices have been successfully used for assaying hepatoprotective activity of curcumin [28], stem bark extract of *Pterocarpus marsupium* [29] and leaf extract of *Atalantia ceylanica* [15]. There is a similarity in liver specific metabolic activities between porcine and human liver cells [30]. Hence liver slices prepared from fresh porcine liver obtained from the slaughter house were used in this study.

**Fig 5:**
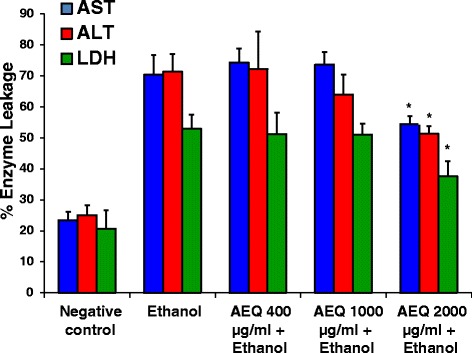
Percentage enzyme leakage of porcine liver slices after 2 h exposure (at 37 °C) to medium (negative control), ethanol and different concentrations of AEQ (400, 1000, 2000 μg/ml) with ethanol. Each value represents mean + SD (*n* = 3). ^*^ *p* < 0.05 when compared to ethanol treated sample with respect to the enzyme assayed. AST = Aspartate transaminase, ALT = Alanine transaminase, LDH = Lactate dehydrogenase

Trial experiments were performed to find the optimum conditions needed and it was found that the minimum concentration of ethanol and exposure time required to induce liver damage was 5 M and 2 h respectively. Ethanol induced toxicity was assessed by leakage of AST, ALT, and LDH enzymes to the medium which indicates the loss of functional integrity of the cell membrane [31]. Percentage AST, ALT, and LDH released in liver slices treated with ethanol (5 M) for 2 h were 70.4 %, 71.3 % and 53.1 % and for untreated liver slices (negative control), the values were 25.0 %, 23.4 % and 20.7 % respectively (Fig. 5). It was observed that there was a significant (*p* < 0.05) reduction in percentage release of ALT, AST and LDH after treatment with AEQ at a concentration of 2 mg/ml (Fig. 5). The percentage release of the liver enzymes from the liver tissue co-exposed with ethanol and the plant extract (2 mg/ml) were markedly reduced and the values were 51.3 %, 54.4 % and 37.6 % for ALT, AST and LDH respectively. This confirms the protective effect of AEQ caused against ethanol induced liver damage.

**Fig 6:**
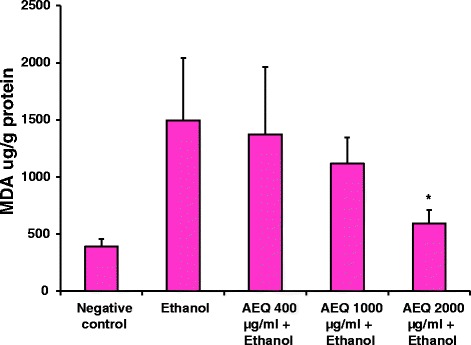
MDA equivalents formed in liver tissue homogenates after exposure to different conditions for 2 h incubation at 37 °C. Each value represents mean + SD (*n* = 3). ^*^ *p* < 0.05 when compared to ethanol (5 M) treated sample. MDA = Malondialdehyde

Free radicals generated on ethanol consumption increases the cellular oxidative stress and results in damages to various cellular components eventually forming products such as lipoperoxides, conjugated dienes and malondialdehyde (MDA). This results in simultaneous reduction of endogenous antioxidant levels such as vitamin E, glutathione, superoxide dismutase, glutathione reductase etc. in tissues [32, 33]. Treatment of liver tissue with ethanol caused significant increase in the amount of lipid peroxides generated (*p* < 0.05) compared to the negative control (Fig. 6). However it was observed that the lipid peroxides formed in the ethanol treated liver slices were significantly (*p* < 0.05) reduced by AEQ at a concentration of 2 μg/ml (Fig. 6). The values for lipid peroxides in the negative control, ethanol induced liver toxicity and treated with plant extract in ethanol induced toxicity were 390 ± 68, 1496 ± 545 and 594 ± 115 μg MDA equivalents/g of protein respectively (Fig. 6). These results show that phytoconstituents present in AEQ have been able to scavenge lipid peroxides generated from 1-hydroxyethyl radical, an oxidative product of ethanol. AEQ possessing potent antioxidant capacity as evident by the DPPH, NO, Hydroxyl radical scavenging and FRAP assays undoubtedly has led towards preventing the formation of such oxidative products. The plant extracts alone in KRHB did not show any toxicity on liver tissue over the concentrations we studied. In a recent study carried out by Nazari and coauthors (2015), it has been found that ethanolic root extract of *Taraxacum Syriacum Boiss* can exert hepatoprotective activity against liver toxicity induced by acetaminophen (APAP) intoxication in rats [34]. The authors believe that the mechanism through which the extract protects the liver from the oxidative stress caused by APAP is due to its antioxidant activity. It was suggested that the phenolic compounds present in the extract are responsible to exert the antioxidant mechanism through scavenging several reactive oxygen species. Such inferences can be made on the hepatoprotection caused by AEQ along with its high phenolic composition.

### Membrane stabilization activity

**Fig 7:**
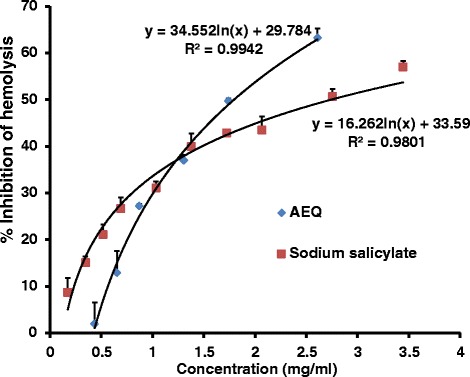
The dose response curves for percentage inhibition of hemolysis by AEQ and sodium salicylate. The results are presented as mean + SD of three independent experiments

The erythrocyte membrane is analogous to the lysosomal membrane [18]. A plant extract which stabilizes the lysosomal membrane suggests that it limits the release of lysosomal enzymes from lysosomes in activated neutrophils into the surrounding tissue. The non steroidal drugs act either by inhibiting these lysosomal enzymes or by stabilizing the lysosomal membrane [18]. The IC_50_ values obtained in HEHA were 1.79 ± 0.04 mg/ml and 2.55 ± 0.08 mg/ml for AEQ and sodium salicylate respectively. This suggests the ability of AEQ to prevent hypotonic solution induced human erythrocyte membrane rupture is higher with respect to sodium salicylate. The dose response curves for the plant extract and sodium salicylate are illustrated in Fig. 7. Antioxidants possess profound ability to prevent the generation of ROS/RNS and enhance the resistance of biological target towards its attack [35]. This might be a possible reason for the suppression of the leakage of intracellular enzymes from liver tissue in addition to the membrane stabilizing activity induced by the phytochemicals present in AEQ. A research study was carried out recently by Shameela and coauthors (2015) to determine hepatoprotective activity of ethanolic extract of the whole plant of *Boerhaavia diffusa* against hepatitis induced by the administration of isoproterenol in Wistar rats [36]. The authors conclude that two possible reasons for the overall hepatoprotective effect of *B. diffusa* is due to a counteraction of free radicals by its antioxidant property or by its membrane stabilizing which helps to protect hepatocellular membrane against oxidative damages. Therefore similar deduction can be made of the potent antioxidants abundant in AEQ and its inherent membrane stabilizing effect.

## Conclusion

Our findings suggest that the decoction prepared from *E. quinquangulare* has the potential to act as a strong antioxidant, hepatoprotective and a membrane stabilization agent. Mechanisms of membrane stabilization and free radical scavenging activity most possibly may contribute towards potent hepatoprotective activity possessed by the plant extract hence justifying its application in traditional medicinal system in Sri Lanka to treat liver ailments. However, further in vivo work including clinical trials is required to determine the synergistic and holistic effect caused by this decoction on the body.

## Additional file

Additional file 1:
**IACUC Principles and Procedures of Animal Care and Use.** (PDF 270 kb)
